# Supplementary material for: Complex‐centric proteome profiling by SEC‐SWATH‐MS
Source: Mol Syst Biol. 2019 Jan 14;15(1):e8438. doi: 10.15252/msb.20188438 (PMC6346213; doi:10.15252/msb.20188438)
Supplement: Supplementary file 8 — Dataset EV7 [file MSB-15-e8438-s008.zip › feature_plots_string/Q9NXR7.pdf]

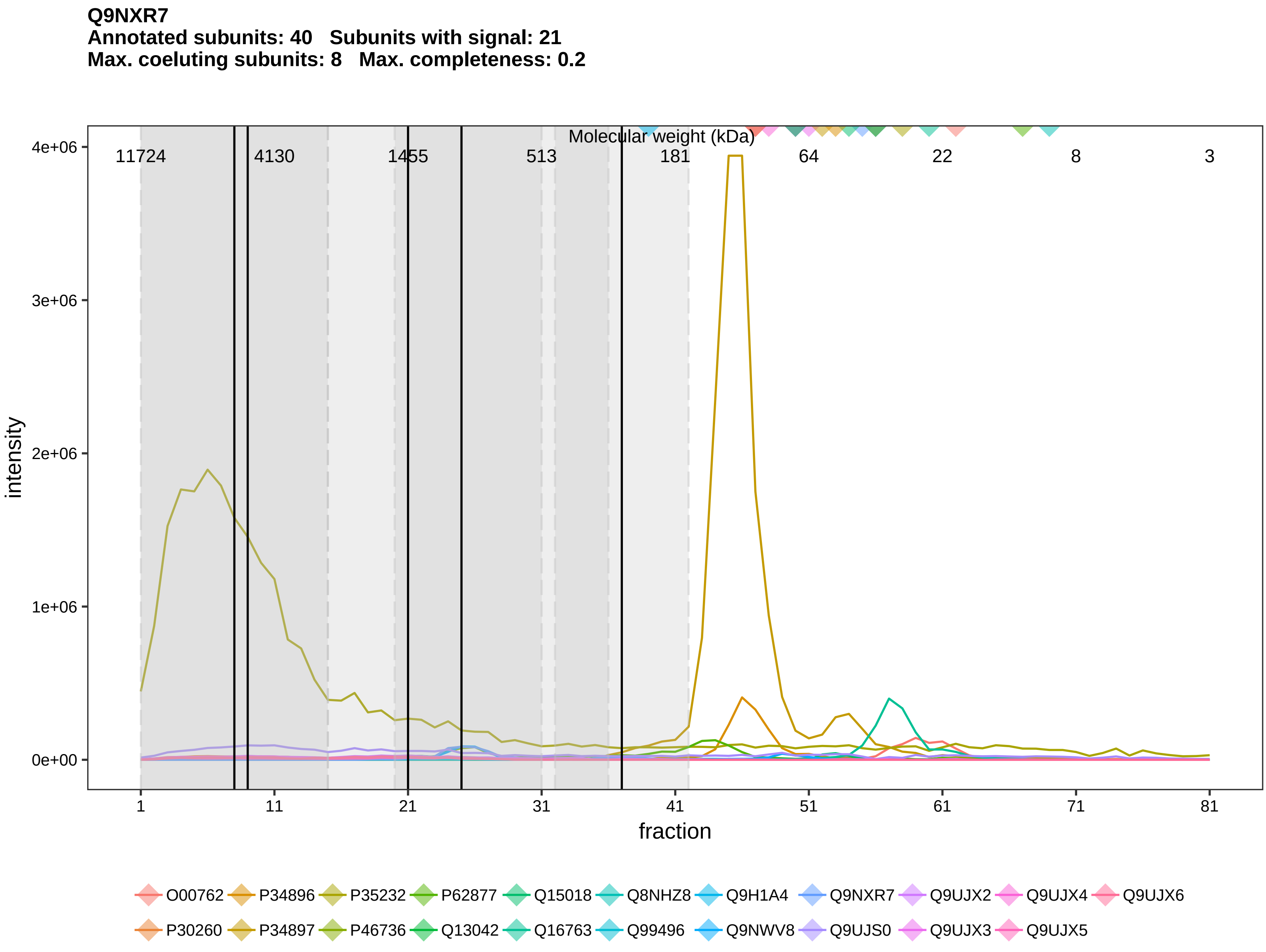

Q9NXR7
Annotated subunits: 40 Subunits with signal: 21
Max. coeluting subunits: 8 Max. completeness: 0.2
Molecular weight (kDa)
11724
4130
1455
513
181
64
22
8
3
intensity
4e+06
3e+06
2e+06
1e+06
0e+00
fraction
1
11
21
31
41
51
61
71
81
O00762
P34896
P35232
P62877
Q15018
Q8NHZ8
Q9H1A4
Q9NXR7
Q9UJX2
Q9UJX4
Q9UJX6
P30260
P34897
P46736
Q13042
Q16763
Q99496
Q9NWV8
Q9UJS0
Q9UJX3
Q9UJX5
